# Beyond GLP-1: Charting the Future of Peptide Therapeutics in Metabolic Disease: Short title: Beyond GLP-1 Peptide Therapeutics

**DOI:** 10.31661/gmj.v15i0.4342

**Published:** 2026-08-26

**Authors:** Babak Pezeshki, Mojtaba Farjam, Aliasghar Karimi

**Affiliations:** Editorial Board of Galen Medical Journal

**Keywords:** GLP-1, Obesity, Peptide Therapeutics, Multi-agonists, Precision Medicine, Metabolic Disease

## Abstract

Glucagon-like peptide-1 (GLP-1)-based therapies have transformed obesity and type 2 diabetes care, establishing pharmacological weight reduction as a route to broader cardiovascular, renal, heart-failure, and metabolic benefit. Their success, however, should be viewed as a platform rather than a therapeutic endpoint. Dual- and triple-receptor agonists, amylin-based combinations, and related endocrine biologics are expanding the field from appetite suppression toward coordinated control of energy balance, glycemia, hepatic metabolism, and organ risk. The central challenge is consequently shifting from maximal agonism to optimized polypharmacology: selecting receptor combinations, potency ratios, and treatment strategies for predefined clinical objectives and patient phenotypes. Precision phenotyping and AI-enabled molecular design may accelerate this transition, but durable benefit will also depend on preservation of physical function, long-term safety, sustainable maintenance, and equitable access. The next generation of metabolic therapeutics should therefore be judged not by maximal short-term weight loss alone, but by durable health gain across organs, function, quality of life, and populations.

## The GLP-1 Benchmark

The arrival of Glucagon-like peptide-1 (GLP-1) receptor agonists has changed both clinical practice and the therapeutic expectations surrounding metabolic diseas. Semaglutide demonstrated that double-digit weight reduction could be achieved pharmacologically in many adults with obesity, while tirzepatide showed that combined glucose-dependent insulinotropic polypeptide (GIP) and GLP-1 receptor agonism could extend glycemic and weight efficacy [1, 2].

More importantly, the value of GLP-1-based treatment is no longer defined by body weight alone. Semaglutide has reduced major cardiovascular events in people with overweight or obesity and established cardiovascular disease, improved symptoms and physical limitations in obesity-related heart failure with preserved ejection fraction, and reduced clinically important kidney outcomes in type 2 diabetes with chronic kidney disease [3, 4, 5].

These outcomes establish a new benchmark. Yet they should not define the ceiling of endocrine pharmacotherapy.

The central question for the next decade is not simply whether newer agents can produce a larger mean reduction in body weight, but whether increasingly sophisticated hormone-based therapies can be matched to the biological drivers, complications, and long-term priorities of individual patients.

## Beyond Single-Receptor Agonism

GIP provides the clearest clinical proof that rationally balanced multi-receptor pharmacology can improve on selective GLP-1 receptor agonism. In a head-to-head trial in type 2 diabetes, tirzepatide produced greater reductions in glycated hemoglobin and body weight than semaglutide 1 mg [6]. The principle has since expanded to glucagon-containing molecules. In phase 2 obesity testing, retatrutide - a single agonist targeting GIP, GLP-1, and glucagon receptors - produced weight reduction of approximately one-quarter of baseline body weight at the highest dose [7]. Phase 3 data in type 2 diabetes have subsequently confirmed substantial glycemic and weight effects, supporting the translational potential of triple agonism while longer-term obesity and outcome data continue to mature [8].

Survodutide, a glucagon/GLP-1 dual agonist, has also progressed to phase 3. Its randomized phase 3 obesity trial confirmed greater weight reduction than placebo, but also highlighted how tolerability, dose escalation, and actual treatment exposure can shape the performance of a multi-receptor drug [9]. This is an important lesson for the field: receptor multiplicity is not itself a therapeutic endpoint.

Amylin biology offers a complementary route. Cagrilintide targets satiety and meal termination through mechanisms that overlap only partly with GLP-1 signaling. The phase 3 REDEFINE 1 trial demonstrated clinically meaningful weight reduction with coadministered cagrilintide and semaglutide, supporting the broader concept that future therapies need not be confined to increasingly complex incretin receptor agonism [10].

The broader endocrine-biologic landscape also includes fibroblast growth factor 21 (FGF21) analogs. Pegozafermin and efruxifermin have produced encouraging effects on histological and metabolic endpoints in steatohepatitis with fibrosis [11, 12]. These agents are better viewed as engineered endocrine proteins than classical peptide drugs, but their development is conceptually important: future metabolic treatment may increasingly be designed around organ-specific disease rather than around weight loss alone.

## From Maximal Agonism to Optimized Polypharmacology

The key scientific transition is therefore from maximal agonism to optimized polypharmacology. Adding receptor targets does not automatically improve treatment. Clinical effects depend on relative receptor potency, pharmacokinetics, tissue exposure, receptor occupancy, signaling characteristics, dose escalation, and the balance between efficacy and adverse effects. Two molecules nominally directed at the same receptor set may consequently have very different clinical profiles.

Future development should begin with predefined therapeutic objectives rather than a competition for the largest mean percentage reduction in body weight. A molecule intended primarily for obesity with metabolic liver disease may require a different biological balance from one intended for obesity with advanced cardiovascular or kidney disease. Similarly, the preferred therapy for an older adult at risk of frailty may differ from that for a younger person with severe insulin resistance and preserved physical reserve. This reframing turns multi-agonism from a molecular achievement into a clinical design problem.

## Precision Treatment and AI-Enabled Design

Precision metabolic medicine is a logical extension of this approach, but current evidence should be interpreted cautiously. Pharmacogenomic analyses have identified genetic variants associated with glycemic response to GLP-1 receptor agonists [13]. More recently, a large genome-wide study identified GLP1R variation associated with weight-loss response and GLP1R and GIPR variation associated with treatment-related nausea or vomiting [14]. These findings establish biological heterogeneity, but their effect sizes and current predictive performance do not justify routine genotype-directed prescribing.

Near-term precision treatment is more likely to emerge from integrating clinical phenotype with molecular data. Appetite phenotype, insulin secretory capacity, hepatic steatosis, kidney function, cardiovascular disease, body composition, frailty, previous treatment response, tolerability, and patient preference may together prove more useful than any single biomarker. The objective should be to move from empirical escalation toward rational selection of the therapy most likely to deliver the desired clinical outcome.

Artificial intelligence may accelerate the molecular side of this transition. AlphaFold 3, RFdiffusion, and ProteinMPNN have expanded the computational toolkit for predicting biomolecular interactions and designing protein structures and sequences [15, 16, 17]. Their relevance to metabolic therapeutics is promising but should not be overstated. These systems can generate and prioritize experimentally testable designs; they do not replace receptor pharmacology, toxicology, manufacturability testing, translational physiology, or randomized clinical trials. In this field, AI should shorten the path to better hypotheses rather than lower the evidentiary threshold for a drug.

## Durability, Function, and Safety

Durability remains one of the most important unresolved clinical problems. Withdrawal studies with semaglutide and tirzepatide show substantial weight regain when active treatment is stopped, reinforcing the biological reality that obesity commonly requires long-term management [18, 19]. The relevant research agenda is therefore not whether chronic disease can be treated as a short course, but how long-term therapy can be made more sustainable. Maintenance dosing, de-escalation after target attainment, sequential treatment, rational combinations, and lower-burden regimens deserve prospective evaluation.

The quality of weight loss also requires greater attention. Highly effective GLP-1-based treatment reduces both fat mass and lean tissue. Lean mass measured by imaging is not synonymous with skeletal muscle, and loss of lean tissue should not automatically be equated with clinically important sarcopenia. Nevertheless, muscle strength, physical performance, nutritional adequacy, and bone health become increasingly relevant as the magnitude and speed of weight reduction increase, particularly in older or frail adults [20]. Future trials should incorporate functional outcomes alongside conventional measures of body weight and waist circumference.

Safety assessment must evolve in parallel with pharmacological potency. Gastrointestinal adverse effects remain common across incretin-based therapies, and glucagon-containing or other multi-pathway agents may introduce additional physiological trade-offs. Longer-duration randomized trials, active-comparator studies, and internationally coordinated pharmacovigilance will be essential if treatment begins earlier in life and continues for decades.

## Access is Part of Effectiveness

No metabolic therapeutic revolution can be considered successful if its benefits remain unavailable to most eligible patients. High prices, manufacturing constraints, reimbursement restrictions, and unequal health-system capacity can transform biological efficacy into limited population impact. Recent Iranian commentary has highlighted local manufacturing as one possible route to narrow the access gap, while appropriately emphasizing the need for comparative evidence, quality assurance, and post-marketing surveillance [21].

The policy challenge is global. The World Health Organization’s 2025 guidance on GLP-1 therapies for obesity explicitly identifies affordability, health-system preparedness, long-term evidence, and equity as implementation constraints and calls for strategies that broaden access [22]. Manufacturing expansion, pooled procurement, tiered pricing, voluntary licensing, and transparent prioritization should therefore be considered part of the therapeutic agenda rather than post-approval concerns. For journals and clinicians in middle-income settings, this is especially important: the most sophisticated molecule has limited public-health value if continuity of supply, affordability, and pharmacovigilance cannot be assured.

## Conclusion

GLP-1 receptor agonists have opened a new era in metabolic medicine, but their most important legacy may be the therapeutic platform they created. The next generation of hormone-based therapeutics should not be judged solely by whether it produces greater short-term weight loss. Success will depend on whether receptor biology can be aligned with patient phenotype, organ-specific risk, physical function, long-term safety, and realistic access.

The future is therefore unlikely to belong to a single dominant peptide or receptor combination. It will belong to therapies developed around explicit clinical objectives and evaluated against outcomes that matter over years rather than months. The defining question for the coming decade should no longer be simply how much weight can be lost, but which treatment can deliver the greatest durable health benefit for the individual patient and for the population in which that treatment must be implemented.

## Conflict of Interest

The authors declare no conflicts of interest.

## AI Disclosure Statement

During the preparation of this work, the authors used ChatGPT (OpenAI) to support scientific language editing, manuscript restructuring, and improvement of clarity and readability. After using this tool, the authors reviewed and edited the content as needed and take full responsibility for the content of the publication.
